# Vaccine Potential of a Recombinant Bivalent Fusion Protein LcrV-HSP70 Against Plague and Yersiniosis

**DOI:** 10.3389/fimmu.2020.00988

**Published:** 2020-06-12

**Authors:** Ankit Gupta, Bineet Narayan, Subodh Kumar, Shailendra Kumar Verma

**Affiliations:** Microbiology Division, Defence Research and Development Establishment, Gwalior, India

**Keywords:** cellular immune response, humoral immune response, LcrV-HSP70, plague, yersiniosis, *Yersinia enterocolitica*, *Yersinia pestis*, *Yersinia pseudotuberculosis*

## Abstract

To counteract the deadly pathogens, i.e., *Y. pestis, Y. enetrocolitica*, and *Y. pseudotuberculosis*, we prepared a recombinant DNA construct *lcrV-hsp70* encoding the bivalent fusion protein LcrV-HSP70. The *lcrV* gene of *Y. pestis* and *hsp70* domain II DNA fragment of *M. tuberculosis* were amplified by PCR. The *lcrV* amplicon was first ligated in the pET vector using NcoI and BamHI restriction sites. Just downstream to the *lcrV* gene, the *hsp70* domain II was ligated using BamHI and Hind III restriction sites. The in-frame and the orientation of cloned lcrV-hsp70 were checked by restriction analysis and nucleotide sequencing. The recombinant bivalent fusion protein LcrV-HSP70 was expressed in *E. coli* and purified by affinity chromatography. The vaccine potential of LcrV-HSP70 fusion protein was evaluated in formulation with alum. BALB/c mice were vaccinated, and the humoral and cellular immune responses were studied. The fusion protein LcrV-HSP70 induced a strong and significant humoral immune response in comparison to control animals. We also observed a significant difference in the expression levels of IFN-γ and TNF-α in LcrV–HSP70-immunized mice in comparison to control, HSP70, and LcrV groups. To test the protective efficacy of the LcrV–HSP70 fusion protein against plague and Yersiniosis, the vaccinated mice were challenged with *Y. pestis, Y. enterocolitica*, and *Y. pseudotuberculosis* separately. The bivalent fusion protein LcrV–HSP70 imparted 100% protection against the plague. In the case of Yersiniosis, on day 2 post challenge, there was a significant reduction in the number of CFU of *Y. enterocolitica* and *Y. pseudotuberculosis* in the blood (CFU/ml) and the spleen (CFU/g) of vaccinated animals in comparison to the LcrV, HSP70, and control group animals.

## Introduction

The *Yersinia* genus contains three pathogenic species, i.e., *Y. pestis, Y*. *pseudotuberculosis*, and *Y. enterocolitica*, which cause fatal infections to human beings (1). Of these, *Y. enterocolitica* and *Y. pseudotuberculosis* are responsible for Yersiniosis, a self-limiting infection. The bacilli responsible for Yersiniosis are passed on through oral or fecal routes mainly from water, soil, and food (1). The symptoms of Yersiniosis are typically mesenteric lymphadenitis, mild diarrhea, acute gastroenteritis, and reactive arthritis (1, 2).

Plague is a highly lethal and rapid disease caused by *Y. pestis*. This pathogen is responsible for millions of deaths throughout the world. According to the World Health Organization (WHO), the plague is a re-emerging disease, and it remains an important public health issue (3, 4). The Center for Disease Control and Prevention (CDC) has classified *Y. pestis* as a group-3 risk pathogen. Plague is a zoonotic infection, and infected wild rats exist as reservoirs in endemic areas throughout the world. The human population is highly susceptible to *Y. pestis* infection, and the manifestation of the infection is mainly dependent on the route of transmission and infection source. Consequentially, the plague develops in one of the three main clinical forms bubonic, septicaemic, and pneumonic (5). Mostly in nature, *Y. pestis* transmitted to humans accidentally after the bite of an infected flea. However, it can also be transmitted via inhalation of aerosolized plague bacilli (6, 7). Transmission of bacilli after bite of a flea develops into a bubonic form of the disease, which is typically characterized by the rapid dissemination of bacilli into the lymph nodes, and their replication is responsible for the development of swollen buboes, an identifying characteristic of the disease. The bubonic form can develop into septicemic or secondary pneumonic plague if the disease is not treated in time (8, 9).

In this modern world, the intentional use of aerosolized *Y. pestis* is a serious threat because of its high fatality rate and its rapid individual-to-individual transmission competence. For the treatment of plague, antibiotics are available. The effectiveness of these antibiotics has been confirmed in humans as well as in animal models (10, 11). However, according to some reports, multidrug-resistant strains of *Y. pestis* have been isolated (12, 13). By genetic engineering, antibiotic-resistant strains of virulent *Y. pestis* may be engineered by manipulating the plasmids harboring the antibiotic-resistant genes (13, 14). In these circumstances, the development of a new generation drugs or vaccines is of the utmost importance to control the disease.

F1 and LcrV are the major vaccine antigens that have been targeted by various scientists to develop a potential vaccine. However, there is no approved vaccine yet. In *Y. pestis*, LcrV is one virulence factor and an essential part of the type III secretion system (T3SS) (15). In continuation of our efforts to develop an anti-plague vaccine, here, we designed a recombinant construct *lcrV*–*hsp70* encoding a fusion protein LcrV-HSP70 of 60 kDa. This recombinant protein was successfully expressed in *E. coli* and purified by immobilized metal affinity chromatography. In order to evaluate the vaccine potential of bivalent fusion protein LcrV-HSP70, Balb/C mice were immunized. The humoral and cellular immune responses were studied, and, ultimately, the protective efficacy against challenges with *Y. pestis, Y. pseudotuberculosis*, and *Y. enterocolitica* were evaluated.

## Materials and Methods

### Ethics Statement

All the protocols for conducting the experiments (MB-44/57/SKV) using BALB/c mice were approved by the Institutional Animal Ethics Committee (IAEC) of Defense Research and Development Establishment (DRDE). This study was carried out in strict accordance with recommendations from the Care and Use of Laboratory Animals committee for the purpose of control and supervision of experiments on animals (CPCSEA), Govt. of India.

### Bacterial Strains, Plasmids, and Reagents

*Escherichia coli* bacterial strains, i.e., DH5α and BL21 (DE3), were procured from Invitrogen, USA. The plasmid pET28a was purchased from Novagen, USA. The bacterial strains, i.e., *Y. pestis* (S1 strain), *Y. pseudotuberculosis* (A87 strain), and *Y. enterocolitica* (O:8 serotype) were collected from DRDE repository. All the challenge experiments using *Y. pestis* were conducted in a biosafety level-3 facility at DRDE, Gwalior.

### Cloning of *lcrV–hsp70* Construct in pET Vector

*Y. pestis* (S1 strain) was grown on a Brain Heart Infusion (BHI) agar plate at 28°C for 48 h. In order to isolate the genomic DNA, one colony from the BHI agar plate was picked up, inoculated in BHI broth (5 ml), and grown at 28°C for 48 h. The culture was pelleted at 10,000 × g for 1 min. The genomic DNA was isolated using commercially available kit (Qiagen, Germany). To clone the *lcrV-hsp70* construct, the *lcrV* gene of *Y. pestis* was amplified by PCR using the forward 5′-ATACCATGGGCATGATTAGAGCCTACGAACAAAAC-3′and reverse 5′-TAGGATCCTTTACCAGACGTGTCATCTAGCA-3′ primers. The PCR amplicon was cloned in the pET28a vector using the *NcoI* and *BamHI* restriction sites (underlined nucleotide sequence). Similarly, the *hsp70* domain (II) of *M. tuberculosis* was also amplified using forward 5′-TAGGATCCGAGAAGGAGCAGCGAATCCTG-3′ and reverse 5′-TAAAGCTTCGGGGTAACATCAAGCAGCAG-3′ primers. This amplified DNA product encoding HSP70 (domain II) was ligated to just downstream to the cloned *lcrV* gene using the *BamHI* and *Hind III* restriction sites (underlined nucleotide sequence) in the same plasmid. The pET plasmid carrying the in-frame of the *lcrV-hsp70* DNA construct was transformed into DH5α cells. The positive transformants were selected on LB-agar plates containing kanamycin (50 μg/ml).

### Preparations of Recombinant Proteins

#### Expression

One of the positive clones encoding the bivalent fusion protein LcrV-HSP70 was inoculated in 5 ml of LB broth containing 50 μg/ml of kanamycin. As reported earlier, LcrV of *Yersinia pestis* and HSP70 domain II of *M. tuberculosis* were also expressed individually (16). In brief, one full loop from stock cultures corresponding to LcrV and HSP70 (stored at −80°C) was inoculated individually into 5 ml of LB broth containing kanamycin. All three cultures were grown individually at 37°C at 200 rpm overnight. The next day, 5 ml LB broth was inoculated with 1% (v/v) individually to each overnight grown culture, and all cultures were grown at 37°C. The cultures were induced with IPTG and grown further for 4 h. One milliliter of uninduced culture was collected individually from each culture prior to adding IPTG. The induced and uninduced cultures were centrifuged at 10,000 × g for 1 min. For SDS-PAGE analysis, the cultures were lysed in 1X sample buffer (0.313 m Tris-HCL; pH 6.8; 50% glycerol; 10% SDS and 0.05% bromophenol blue; 100 mM DDT), and they were electrophoresed on SDS-PAGE.

#### Purification

The recombinant proteins LcrV-HSP70, LcrV, and HSP70 fused with histidine tags were purified under native conditions using Ni-NTA columns (Qiagen, Germany). One full loop of cultures corresponding to LcrV-HSP70, LcrV, and HSP70 were inoculated individually into 5 ml of LB broth containing 50 μg/ml kanamycin, grown for overnight at 37°C at 200 rpm. The next day, 500 ml of LB broth was inoculated individually with 1% overnight grown culture for each protein and grown at 37°C at 200 rpm. Cultures were induced with IPTG and grown further for 4 h. All the cultures were pelleted individually at 8,000 × g for 10 min at 4°C. Each pellet was suspended individually in 20 ml of lysis buffer (50 mM NaH_2_PO_4_, 250 mM NaCl; 10 mm imidazole; pH 8.0). The suspended pellets were sonicated, centrifuged at 20,000 × g for 30 min at 4°C, and supernatants were separated individually. Three Ni-NTA columns were prepared for each protein. Each column was equilibrated with lysis buffer (~10 ml). The supernatant of individual protein was poured on to the Ni-NTA column and allowed to pass. Each column was washed with 50 ml of wash buffer containing 50 mM NaH_2_PO_4_, pH-8.0; 250 mM NaCl; 30 mM imidazole. The proteins, LcrV-HSP70, LcrV, and HSP70, were eluted individually by applying 15 ml of elution buffer (50 mM NaH_2_PO_4_, 250 mM NaCl; 200 mM imidazole; pH 8.0). The collected fractions for each protein were analyzed by SDS-PAGE. The fractions containing each purified protein were pooled individually and subjected to dialysis by gradually lowering the salt concentration against dialysis buffer (50 mM NaH_2_PO_4_; 50 mm NaCl; pH 8.0). The **c**oncentration of each purified protein was estimated using a BCA kit (Sigma Aldrich, USA). The endotoxin contents were measured in each purified recombinant protein using Limulus Amoebocyte Lysates (LAL) QCL-1000 kit (Cambrex Biosciences, USA) as per the instructions.

#### Western Blot

All three prepared recombinant proteins, i.e., LcrV, HSP70, and LcrV-HSP70, were analyzed by SDS-PAGE for their purity, as shown in **Figure 2Da**. In order to confirm the presence of proteins of interest, an immunoblot experiment was performed. The recombinant proteins were separated by SDS-PAGE and transferred onto the nitrocellulose membrane electrophoretically. The nitrocellulose membrane was blocked overnight at 4°C in 5% skim milk. The membrane was probed with Penta-His HRP conjugate anti-histidine antibody (Cat No./ID: 34460, Qiagen) at a dilution of 1:1,000 in 5% skimmed milk for 1 h at 37°C. The membrane was washed thrice with PBS- containing 0.05% Tween 20 (PBS-T). The reaction was developed in 20 ml of PBS containing 8.8 mM H_2_O_2_ and 10 mg of 3,3′-diaminobenzidine/ml. Development was carried out for 2–3 min until bands of the desired intensity appeared, and thereafter the membrane was washed.

### Animal Immunization

To test the protective efficacy and immune responses of the bivalent fusion protein LcrV-HSP70, 5-week-old female BALB/c mice were taken from DRDE animal facility. Mice were divided into four batches, and each batch was divided into four groups (10 mice/group): the control group, HSP70 group, LcrV group, and the LcrV-HSP70 group (**Figure 3A**). All the groups, except for the control group, were vaccinated subcutaneously with 15 μg/mouse of each vaccine antigen in formulation with alum adjuvant. The animals of batch I, II, and III were used for the evaluation of humoral immune response and protection studies against *Y. pestis, Y. pseudotuberculosis*, and *Y. enterocolitica* challenges, respectively. The animals of batch IV were used for the study of the cellular immune response. The animals of the control group in each batch received sterile PBS only. The animals were immunized on day 0 followed by two boosters on day 14 and 21. In order to study the IgG response, blood was collected on day 0, 21, and 28 (**Figure 3A**).

### Anti-LcrV-HSP70 IgG Response

ELISA plates (Nunc, USA) were coated individually with LcrV, HSP70, and LcrV–HSP70 proteins using 1.0 μg/well and incubated for overnight at 4°C. Plates were washed with 0.05% Tween 20 in PBS (PBS-T), blocked with 3% bovine serum albumin (BSA), and incubated for 2 h at 37°C. After washing the plates thrice with PBS-T, test sera were added in 2-fold serial dilutions in triplicate wells (100 μl/well) and incubated for 1 h at 37°C. After three washes, the wells were probed with rabbit anti-mouse IgG-HRP (Sigma, USA) at 1:15000 dilutions in PBS and incubated for 1 h at 37°C. The ELISA plates were washed, and the reaction was developed with OPD substrate. 2N H_2_SO_4_ was added to terminate the reaction, and the absorbance was read at 490 nm by a multimode reader (Biotek, USA). The IgG titers were represented as log_10_ titers of the highest serial dilution with a mean of OD_490_ value >2-fold OD_490_ value of control serum at the same dilution.

### Cytokines

The animals of batch IV were sacrificed 1 month after the second booster to measure the cytokine levels. The spleens were removed aseptically and homogenized to prepare single-cell suspension. The splenocytes were counted, and 1 × 10^6^ cells/well were seeded in triplicate in a 96-well-plate. The cultures were stimulated with the same vaccine antigen the animal groups were vaccinated with, i.e., HSP70, LcrV, LcrV–HSP70, or ConA (5 μg/ml each). Concanavalin A (Sigma, USA) was used as a positive control. After 48 h, the supernatants of the cultured splenocytes were harvested and stored at −80°C for further use. The expression levels of IFN-γ and TNF-α in the supernatants were measured using ELISA kit (BD Biosciences, USA).

### Bacterial Challenge

To determine the protective efficacy, on day 60 after priming, all the animal groups of batch I were challenged with 100 LD_50_ (1 × 10^5^ CFU/mouse) of *Y. pestis* (S1 strain) via *i.p*. route (16, 17). The infected animals were observed for 20 days for their survival (**Figure 3A**). The protection experiments were repeated thrice.

Similarly, the animals of batch II and III were challenged (1 × 10^8^ CFU/mouse) with *Y. enterocolitica* (O:8 serotype) and (1 × 10^9^ CFU/mouse) and with *Y. pseudotuberculosis* (A87 strain), respectively, via *i.p*. route (**Figure 3A**). To count the bacterial load in the blood and spleen, on days 1, 2, 3, 4, and 5 post-challenge, two mice from each group of batch II and III were sacrificed. Their spleens were removed aseptically and weighed. Each spleen was homogenized, and serial dilutions were made in PBS. One hundred microliter suspensions from each countable dilution were spread on BHI agar plates in duplicate. *Y. pseudotuberculosis* culture plates were incubated at 28°C for 48 h, and the bacterial colonies were counted. *Y. enterocolitica* culture plates were incubated at 37°C for 24 h, and the bacterial colonies were counted. Prior to sacrifice the remaining animals, the blood was collected. Each blood sample was serially diluted in PBS. One hundred microliter from each countable dilution was spread on BHI agar plates in duplicate. The bacterial load (CFU/ml) and (CFU/g) was determined in the blood and spleen, respectively. The protection experiments were repeated thrice, and the results were expressed as the mean log CFU ± SD per group of three experiments.

### Statistical Analysis

Data were analyzed using One Way Analysis of Variance (ANOVA) to compare IgG titers, CFU count, and cytokines response. Log-rank (Mantel-Cox) test was used to generate survival curves using GraphPad Prism 5.0. Statistically significant *p*-values are represented as ^***^ *p* < 0.0001; ^**^p < 0.01; ^*^ *p* < 0.05.

## Results

### Cloning of *lcrV–hsp70* in pET Vector

**Figure 1:**
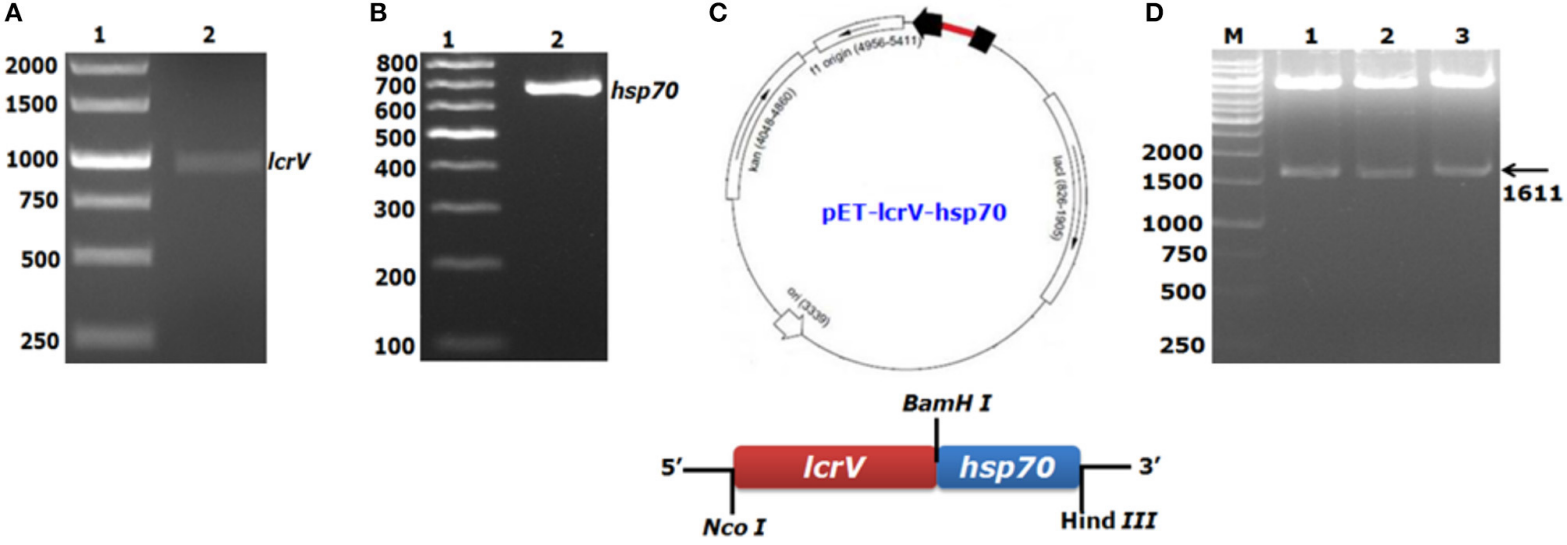
PCR amplification and agarose gel electrophoresis of *lcrV* gene and *hsp70* (domain II) of *M. tuberculosis* and ligation of *lcrV-hsp70* in pET vector: *LcrV* gene **(A)** Lane-1, 1Kb DNA ladder; Lane-2, *lcrV* gene product of 981 bp. **(B)** Lane-1, 1 Kb DNA ladder; Lane-2, *hsp70* gene product of 630 bp. **(C)** The restriction map of cloned *lcrV-hsp70* construct in pET vector. **(D)** Screening of positive clones of *lcrV-hsp70* construct after ligation in pET28 vector, Lane-M, 1 Kb DNA ladder; Lane 1–3, released inserts of *lcrV-hsp70* (1,611 bp) after restriction digestion using NcoI and Hind III restriction enzymes.

For the expression of a bivalent fusion protein LcrV-HSP70, a 981 bp gene of *Y. pestis* encoding LcrV and a 630 bp DNA fragment encoding HSP70 domain II of *M. tuberculosis* were amplified by PCR (Figures 1A,B). The amplicon of the *lcrV* gene was first ligated in pET28a plasmid using NcoI and BamHI restriction sites. Just downstream to *lcrV* gene, a 630 bp fragment of *hsp70* domain II was ligated using BamHI and HindIII restriction sites, as shown in Figure 1C. The pET vector harboring the in-frame of *lcrV-hsp70* DNA construct (*pET-lcrV-hsp70*) was transformed into competent DH5α cells. The cells were grown on LB agar plates containing kanamycin (50 μg/ml). Positive clones were selected by restriction analysis using NcoI and HindIII restriction enzymes. The digested products were analyzed by agarose gel electrophoresis. A DNA insert of 1,611 bp was observed, as shown in Figure 1D. The in-frame and the orientation of the ligated construct were further confirmed by nucleotide sequencing (Chromous Biotech, Bangalore, India).

### Expression and Purification of Recombinant Proteins

#### LcrV-HSP70

**Figure 2:**
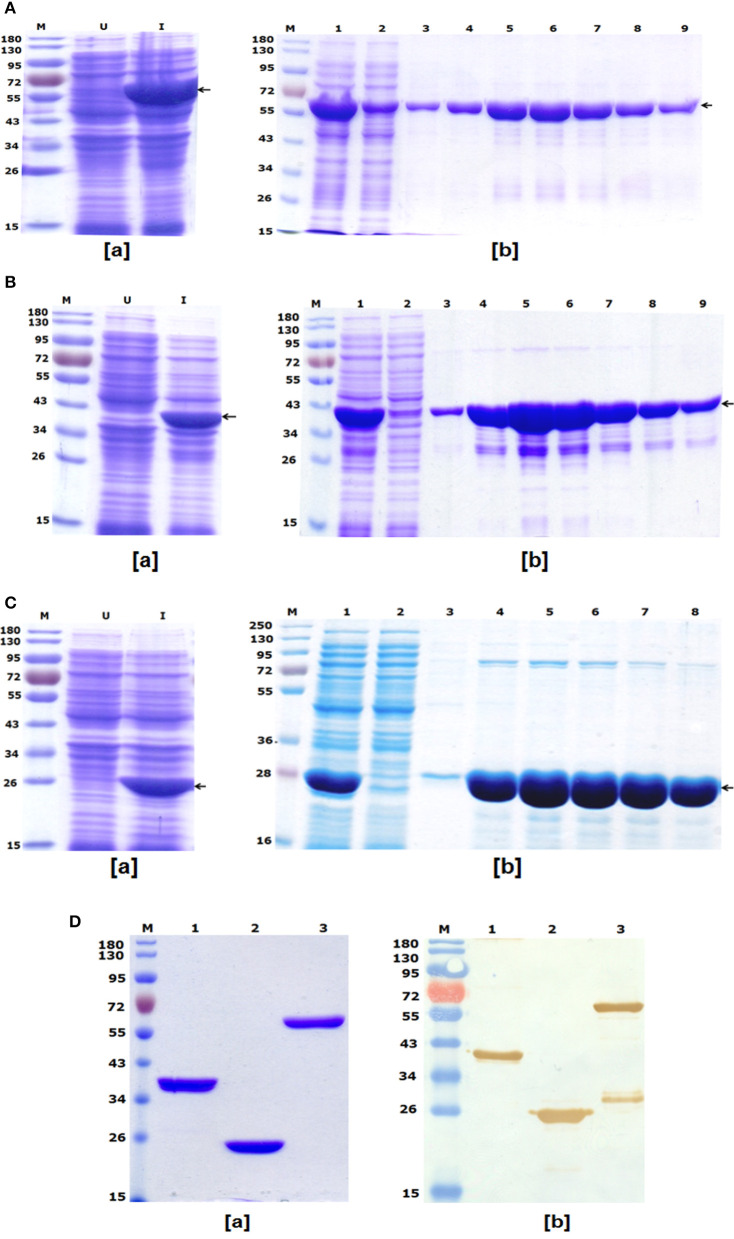
**(A)** Recombinant bivalent fusion protein LcrV-HSP70 expression profile and purification: (a) SDS–PAGE profile of LcrV-HSP70 protein expression; Lane-M, Pre-stained protein marker; Lane-U, Uninduced cell lysate; Lane-I, Induced cell lysate. The arrow at the right side of the SDS-PAGE profile indicates the position of LcrV-HSP70 protein of 60 kDa. (b) Purification of LcrV-HSP70 protein. Lane-M, Protein marker; Lane-1, Cell lysate; Lane-2, Flow through; Lane-3, Wash; Lane 4–9, eluted fractions of LcrV-HSP70 protein. The arrow at the right side of the SDS-PAGE profile indicates the position of LcrV-HSP70 protein of 60 kDa. **(B)** Recombinant LcrV protein expression profile and purification: (a) SDS–PAGE profile of LcrV protein expression; Lane-M, Pre-stained protein marker; Lane-U, Uninduced cell lysate; Lane-I, Induced cell lysate. The arrow at the right side of the SDS-PAGE profile indicates the position of LcrV protein of 37 kDa. (b) Purification of LcrV protein. Lane-M, Protein marker; Lane-1, Cell lysate; Lane-2, Flow through; Lane-3, Wash; Lane 4–9, eluted fractions of LcrV protein. The arrow at the right side of the SDS-PAGE profile indicates the position of LcrV protein of 37 kDa. **(C)** Recombinant HSP70 protein expression and purification: (a) SDS–PAGE profile of HSP70 protein expression; Lane-M, Pre-stained protein marker; Lane-U, Uninduced cell lysate; Lane-I, Induced cell lysate. The arrow at the right side of the SDS-PAGE profile indicates the position of HSP70 protein of 23 kDa. (b) Purification of HSP70 protein. Lane-M, Protein marker; Lane-1, Cell lysate; Lane-2, Flow through; Lane-3, Wash; Lane 4–8, eluted fractions of HSP70 protein. The arrow at the right side of the SDS-PAGE profile indicates the position of HSP70 protein of 23 kDa. **(D)** Western blot analysis of purified recombinant proteins LcrV, HSP70, and LcrV-HSP70: (a) SDS–PAGE profile of purified proteins, Lane-M, Pre-stained protein marker; Lane-1, LcrV, Lane-2, HSP70 and Lane-3, LcrV-HSP70 protein. (b) Western blot analysis of LcrV, HSP70, and LcrV-HSP70 proteins showing reaction with anti-HIS antibody: Lane-M, Pre-stained protein marker; Lane-1, LcrV, Lane-2, HSP70 and Lane-3, LcrV-HSP70 protein.

To express and purify the bivalent fusion protein LcrV-HSP70 (60 kDa), one of the positive clones of *lcrV-hsp70* was transformed into *E. coli* expression host strain BL-21 (DE3). The colonies were selected on LB-agar plates containing kanamycin. One colony was inoculated in 5 ml LB broth, and the culture was induced with 1 mM IPTG. The IPTG-induced and uninduced *E. coli* cell lysates were analyzed by SDS-PAGE, as shown in Figure 2Aa. For purification of LcrV-HSP70 protein, the culture was inoculated in 500 ml LB broth. The cells were pelleted by centrifugation, and the lysis under native conditions revealed the association of LcrV-HSP70 protein in the soluble fraction. The recombinant construct of *lcrV-hsp70* was engineered to carry a histidine tag at the carboxyl terminus of the LcrV-HSP70 protein. The purification was performed under native conditions by affinity chromatography using Ni-NTA resin. All the eluted fractions were analyzed by SDS-PAGE, as shown in Figure 2Ab. The eluted fractions were pooled, dialyzed, and the protein concentration was estimated. The obtained yield of the purified LcrV-HSP70 was 30 mg/L of shake flask culture. The endotoxin level was observed <5 endotoxin unit (EU)/15 μg of each purified protein.

#### LcrV and HSP70

The recombinant LcrV and HSP70 proteins of 37 and 23 kDa, respectively, were also prepared using earlier published protocols (16). Recombinant LcrV protein was expressed and analyzed by SDS-PAGE, as shown in Figure 2Ba. The LcrV protein was purified using native conditions, and the eluted fractions of purified recombinant LcrV were analyzed by SDS-PAGE, as shown in Figure 2Bb. Similarly, recombinant HSP70 protein was expressed and analyzed by SDS-PAGE (Figure 2Ca), and it was purified using native conditions. The eluted fractions of purified recombinant HSP70 was analyzed by SDS-PAGE, as shown in Figure 2Cb. The eluted fractions of each protein were pooled, dialyzed, and analyzed by SDS-PAGE (Figure 2Da). The protein concentrations were estimated and the obtained yield of LcrV and HSP70 were 35 and 25 mg/L of shake flask culture, respectively. The endotoxin level was shown to be <4 endotoxin unit (EU)/15 μg of each purified protein.

### Western Blot

In a Western blot experiment, anti-histidine antibody recognized all three proteins, i.e., LcrV, HSP70, and LcrV-HSP70, corresponding to their molecular weights, as shown in Figure 2Db. In lane-3 of Figure 2Db, we observed an extra band below the main band of LcrV-HSP70. This band is seen only on the Western blot and not on the SDS-PAGE and therefore considered to be a minor contaminant. This may have been the result of some degradation.

### Humoral Immune Response

**Figure 3:**
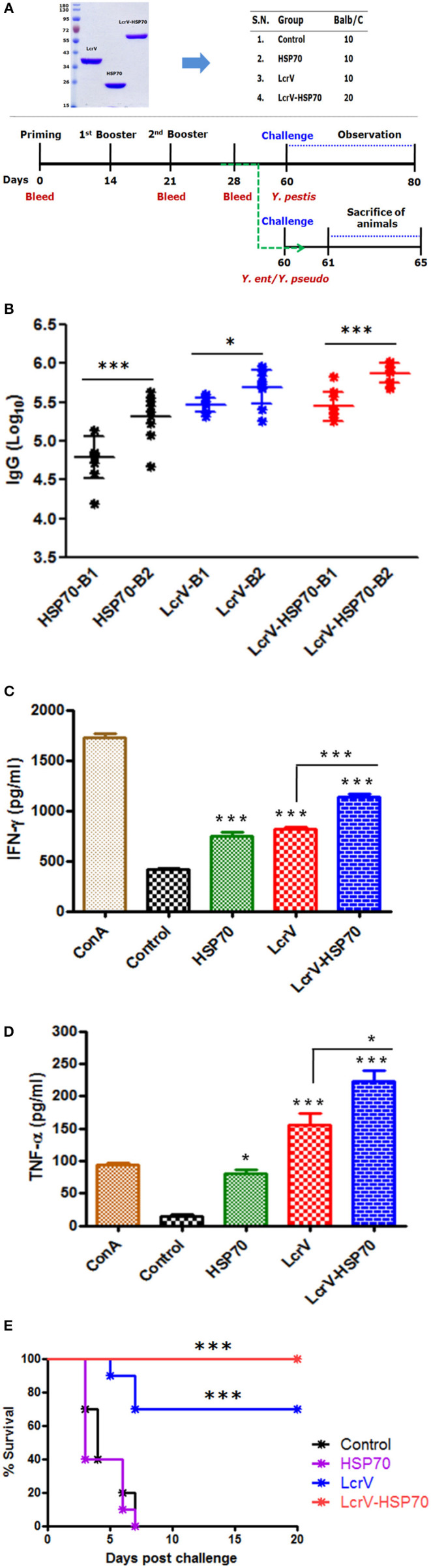
**(A)** Representation of animal groups and schedule of vaccination activities: Animal groups (10 mice/group) for vaccination studies; Vaccine formulations of LcrV, HSP70 and LcrV–HSP70 vaccine antigens with alum. Schematic representation of vaccination schedule, blood collection for humoral and cell-mediated studies, and challenge experiments. **(B)** The ELISA plates were coated by the same antigens, which were used as immunizing antigens. IgG titers were determined by ELISA and represented as log_10_ titers. Serum samples were collected after first and second boosters from vaccinated animal groups, i.e., LcrV, HSP70, and LcrV–HSP70. Analysis was done by one-way ANOVA, ***p < 0.0001; **p < 0.01; *p < 0.05. **(C)** Cytokine profile of IFN-γ and **(D)** TNF-α; the induced levels of cytokines in vaccinated mice were measured in pg/ml, as represented in graphs. Splenocytes were prepared from all the vaccinated and control groups. The cells were induced with the same vaccine antigens as the immunizing antigens, i.e., LcrV, HSP70, and LcrV-HSP70 (5 μg/ml each) and grown for 48 h. All the statistical comparisons were done by one-way ANOVA, ***p < 0.0001; **p < 0.01; *p < 0.05. **(E)** Recombinant bivalent fusion protein LcrV–HSP70 imparts protection against challenge with *Y. pestis*. The immunized and control group mice were challenged against 100 LD_50_ of *Y. pestis*. All the animals were observed for 20 days after the challenge for their survival. Log-rank (Mantel-Cox) test was used to compare the survival against plague infection amongst different vaccinated groups (***p < 0.0001).

In order to test the humoral immune response evoked by LcrV, HSP70, and LcrV-HSP70, IgG endpoint titers were determined by ELISA in the sera taken 7 days after first and second boosters, respectively. The IgG titers were represented as log_10_ titers after first and second booster sera. There was a significant difference in the IgG titer in the sera collected after first and second boosters for each antigen, as shown in the Figure 3B. However, there was no significant difference in the IgG titers in the vaccinated sera of LcrV and LcrV-HSP70.

### Cellular Immune Response

The expression levels of IFN-γ and TNF-α in the collected supernatants of splenocytes were measured. We observed a significant difference in the expression levels IFN-γ (Figure 3C) and TNF-α (Figure 3D) in LcrV, HSP70, and LcrV-HSP70 vaccinated mice in comparison to the control group. ConA was used to induce the spleen cells of all the groups, and it responded non-specifically. There was a significant difference in the expression of IFN-γ (^***^P < 0.0001) and TNF-α (^*^P < 0.05) in the LcrV-HSP70 vaccinated group in comparison to LcrV and HSP70 alone (Figures 3C,D).

### Protection Studies

All the animals of batch I were challenged against 100 LD_50_ of *Y. pestis*. The survival of the infected animals was observed for 20 days after the challenge. There was 100% survival in the LcrV-HSP70 vaccinated group, whereas a 70% survival rate was observed in the LcrV vaccinated group. No protection was observed in the control and HSP70 groups. A Log-rank (Mantel-Cox) test was used to compare the survival amongst different vaccinated groups (^***^P < 0.0001) (Figure 3E).

**Figure 4:**
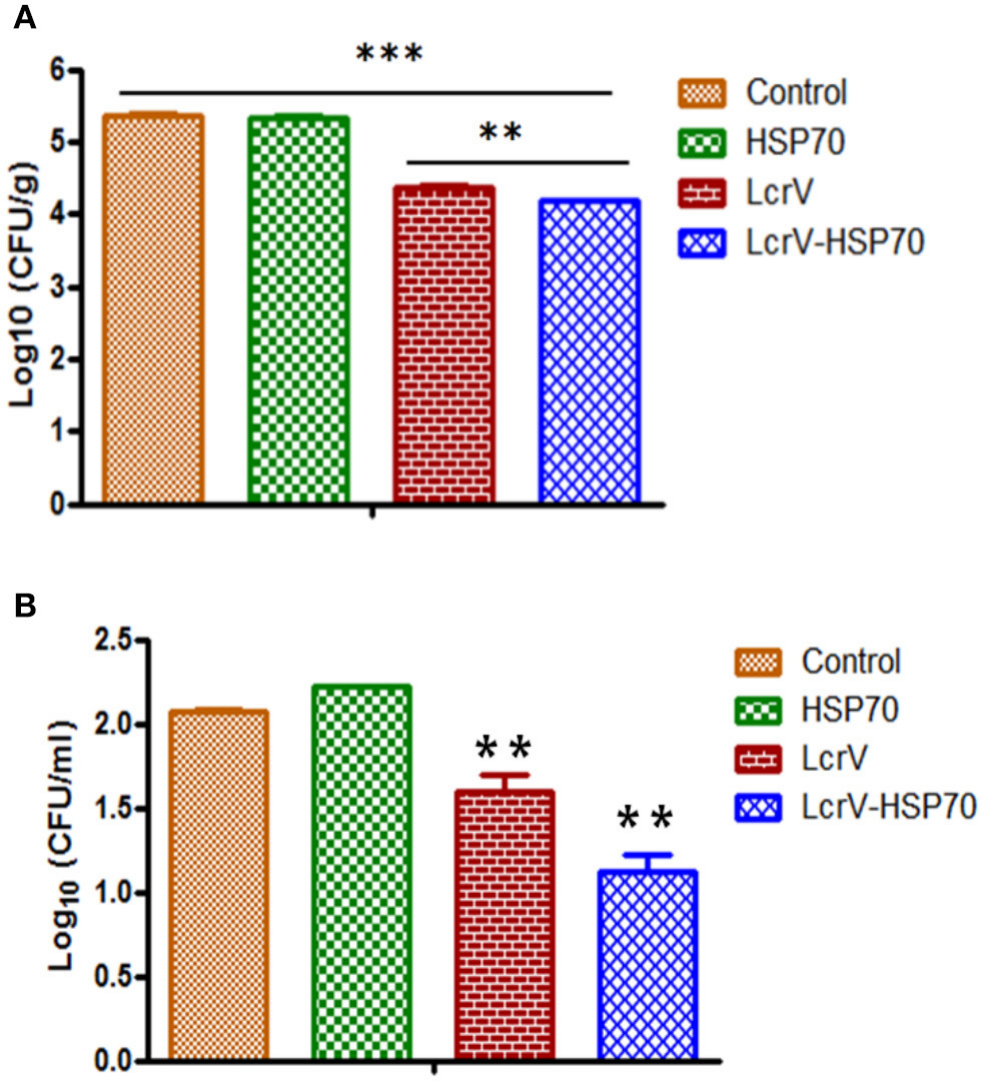
**(A)** CFU count in the spleens of *Y. enterocolitica*-challenged mice. Culture plates were incubated at 37°C for 24 h, and the bacterial colonies were counted. The number of bacteria in the spleens is represented as the mean log CFU ± SD per group. Protection was found statistically significant in LcrV-HSP70 vaccinated group when compared to LcrV (**p < 0.01), HSP70 and control group (***p < 0.0001). **(B)** CFU count in the blood of *Y. enterocolitica* challenged mice: *Y. enterocolitica* culture plates were incubated at 37°C for 24 h and the bacterial colonies were counted. The number of bacteria in the blood is represented as the mean log CFU ± SD per group.

**Figure 5:**
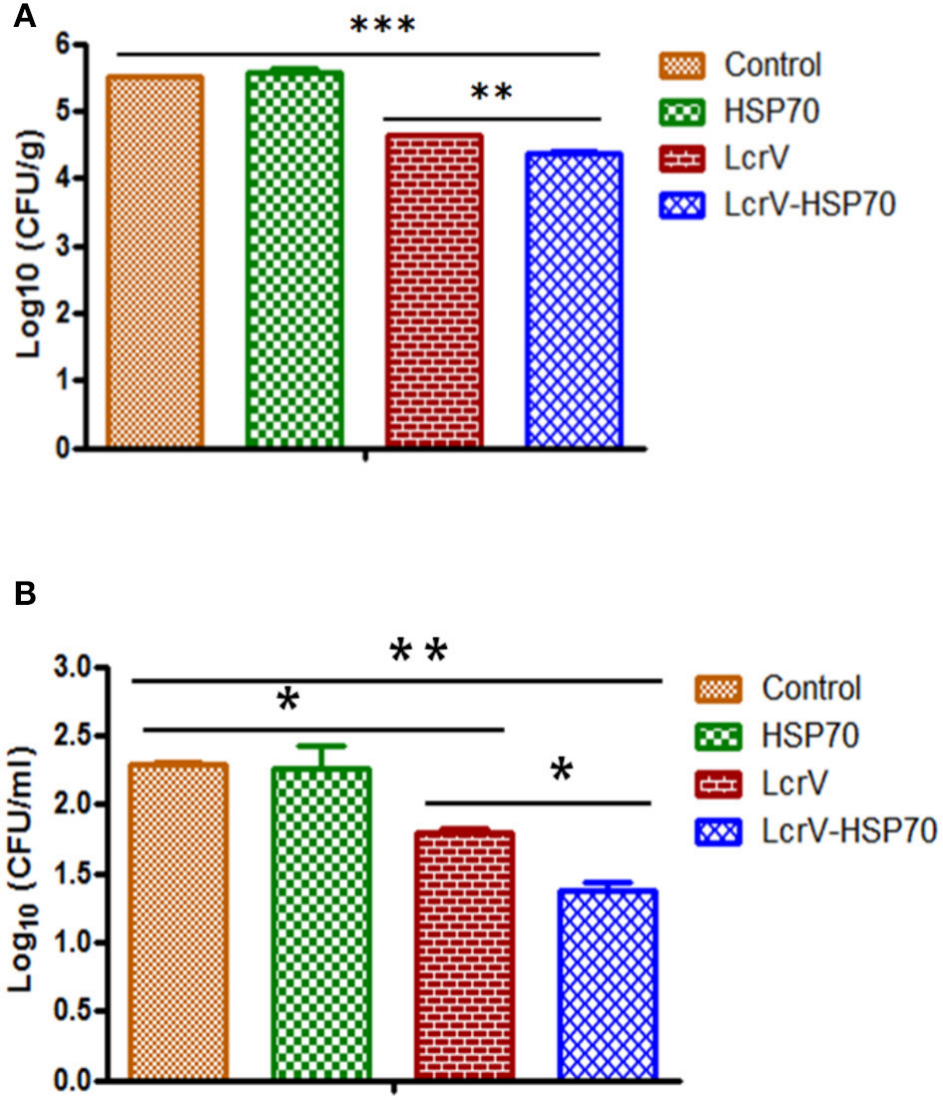
**(A)** CFU count in the spleens of *Y. pseudotuberculosis*-challenged mice. Culture plates were incubated at 28°C for 48 h, and the bacterial colonies were counted. The number of bacteria in the spleens is represented as the mean log CFU ± SD per group. Protection was found statistically significant in LcrV-HSP70 vaccinated group when compared to LcrV (**p < 0.01), HSP70 and control group (***p < 0.0001). **(B)** CFU count in the blood of *Y. pseudotuberculosis* challenged mice: *Y. pseudotuberculosis* culture plates were incubated at 28°C for 48 h, and the bacterial colonies were counted. The number of bacteria in the blood is represented as the mean log CFU ± SD per group (*p = 0.05).

The animals infected with *Y. enterocolitica* and *Y. pseudotuberculosis* of Batch II and III, respectively, were sacrificed to determine the bacterial loads. The level of infection was evaluated by determining CFU count in the blood (CFU/ml) and spleens (CFU/g). The control animal groups of each batch had the highest bacterial counts. The CFUs were counted on day 1, 2, 3, 4, and 5 of post-challenge (Figure S1). On day 2 after challenge, there was a significant reduction in CFUs of *Y. enterocolitica* (Figure 4) and *Y. pseudotuberculosis* (Figure 5) in LcrV-HSP70 vaccinated mice in comparison to LcrV and HSP70 vaccinated animals. In *Y. enterocolitica* challenged mice, the protection unit of LcrV-HSP70 was 1.1905, whereas the protection unit of LcrV and HSP70 were 0.9875 and 0.0215, respectively, in the spleen. In the blood, the protection units of LcrV-HSP70, LcrV, and HSP70 were observed to be 0.9465, 0.468, and 0 respectively (Table 1). In *Y. pseudotuberculosis* challenged mice, the protection unit of LcrV-HSP70 was 0.984, whereas the protection unit of LcrV and HSP70 were 0.886 and 0, respectively, in the spleen. In the blood, the protection unit of LcrV-HSP70, LcrV, and HSP70 were observed to be 0.9815, 0.49, and 0.027, respectively, (Table 2). We did not find any CFU on day 4 in the spleen and on day 3 in the blood. Units of protection were obtained by subtracting the mean log CFU of the vaccinated group from the mean log CFU of the control group.

**Table 1:** Protection on day 2 post-challenge in LcrV-HSP70 vaccinated mice against *Y. enterocolitica*.

**Groups**	**Log_**10**_ CFU *Y. enterocolitica* (Spleen)**	**Unit of protection**	**Log_**10**_ CFU *Y. enterocolitica* (Blood)**	**Unit of protection**
Control	5.365 ± 0.01343	–	2.0695 ± 0.013435	–
HSP70	5.344 ± 0.01060	0.0215	2.2170 ± 0.235467	–
LcrV	4.378 ± 0.05091	0.9875	1.6015 ± 0.063851	0.468
LcrV-HSP70	4.175 ± 0.01060	1.1905	1.1230 ± 0.053460	0.9465

*The number of bacteria in the spleens (CFU/spleen) is represented as the mean log CFU ± SD per group. The unit of protection was determined by subtracting the mean log CFU of the immunized group from the mean log CFU of the control group*.

**Table 2:** Protection on day 2 post-challenge in LcrV-HSP70 vaccinated mice against *Y. pseudotuberculosis*.

**Groups**	**Log_**10**_ CFU *Y. pseudotuberculosis* (Spleen)**	**Unit of protection**	**Log_**10**_ CFU *Y. pseudotuberculosis* (Blood)**	**Unit of protection**
Control	5.511 ± 0.0	–	2.2895 ± 0.016263	–
HSP70	5.574 ± 0.06929	–	2.2625 ± 0.235467	0.027
LcrV	4.625 ± 0.02121	0.886	1.7995 ± 0.030406	0.49
LcrV-HSP70	4.527 ± 0.03676	0.984	1.371 ± 0.097581	0.9185

*The number of bacteria in the spleens (CFU/spleen) is represented as the mean log CFU ± SD per group. The unit of protection was determined by subtracting the mean log CFU of the immunized group from the mean log CFU of the control group*.

## Discussion

Despite continuous and dedicated efforts by the scientific community, an ideal plague vaccine is yet to come. Many plague vaccine formulations based on the live attenuated and killed whole-cell of *Y. pestis* have been developed. Despite having ethical issues and safety concerns, i.e., high fever, headache, and inflammation at the site of injection, these formulations are in use in some countries (18). The main limitations of these vaccine formulations are partial or incomplete protective efficacy, poor memory, and repeated boosters (9, 19, 20). In the recent past, many reports have been published on F1/LcrV based vaccine formulations. The F1/LcrV vaccine antigens adjuvanted with alum induce robust humoral immune responses and impart 100% protection in a mouse model (16, 21–24) with no side effects in humans (25). F1/LcrV antigen-based vaccine formulations protect cynomolgus macaques against aerosolized *Y. pestis* but failed to protect African Green monkeys (26, 27). The immune mechanisms responsible for this poor and inconsistent protection in the African Green monkey model yet to be analyzed. To improve the efficacy of these vaccine formulations by inducing stronger cellular immunity might be the best solution. A number of strategies are in development to advance the efficacy of F1/LcrV antigens, e.g., use of novel and competent adjuvants (28–30), vaccine antigen delivery systems (31, 32), and genetically modified antigens (33). To counteract the pathogen effectively, addition of one or more vaccine antigens might be considered which can significantly augment the immune response.

In the recent years, the efficacy of F1/LcrV based vaccines have been improved by adding molecular adjuvants, i.e., F1/V adjuvanted with flagellin (Flagellin/F1/V) for phase I safety and immunogenicity trial in healthy adult volunteer (34); the recombinant flagellin of *Salmonella typhi* also improved the protective potential of YopE (35); the SA-4-1BBL adjuvant, a strong inducer of the Th1 response, improved the vaccine potential of F1-LcrV (30); and the HSP70 domain II of *M. tuberculosis* augments the humoral and cellular immune response of F1/LcrV vaccine (16, 24). HSP70 proteins have been characterized to immunomodulate the immune response of vaccine antigens (36–42). The domain II of heat shock protein HSP70(II), when formulated with vaccine antigen/s, evoked the T-cell responses. A bivalent fusion protein ovalbumin-HSP70(II) induces CD8 cytotoxic T lymphocytes specific to ovalbumin (36). HSP70(II) of *M. tuberculosis* modulated the immune responses of vaccine antigen p24 of HIV-1 (36). The amino acids (359–610) of the carboxy-terminus of HSP70 evoke the expression of IFN-γ, IL-2, and TNF-α and a high titer of IgG2a and IgG3 antibodies (43).

Earlier, we have also characterized the potential role of HSP70(II) of *M. tuberculosis* to augment the vaccine efficacy of F1/LcrV in a mouse model (16, 24). Our findings showed that HSP70(II) improved the cellular immune response (IL-2, IFN-γ, and TNF-α) of F1+LcrV+HSP70(II)-vaccinated mice. It also enhanced the IFN-γ secreting CD4^+^ and CD8^+^ T cells. Earlier, we developed a recombinant trivalent fusion protein F1-LcrV-HSP70(II) and evaluated as a vaccine candidate against plague in a mouse model (24). We experienced difficulties in the expression and purification of this trivalent fusion protein in *E. coli*. The expression was not found to be optimal, making inclusion bodies during expression. Hence the protein can only be purified under denaturing conditions. Moreover, this trivalent fusion protein is highly degradable at room temperature. In this study, we prepared a recombinant bivalent fusion protein LcrV-HSP70, and we evaluated its vaccine potential in BALB/c mice. The expression of the bivalent fusion protein is up to the mark in *E. coli*, and this protein does not make inclusion during the expression. The bivalent protein can be easily purified under native conditions to the optimum yield. All the three antigens, i.e., LcrV-HSP70, LcrV, and HSP70, were adjuvanted individually with alum, and the animals were vaccinated. There was a significantly higher anti-LcrV IgG titer in the sera of LcrV-HSP70 vaccinated mice in comparison to HSP70 and control group. No significant difference was observed in the anti-LcrV IgG titer between LcrV alone and fusion protein LcrV-HSP70 vaccinated sera. However, there was a significant difference in the IgG titers in the sera after first and second booster. We also observed a significant difference in the expression of IFN-γ and TNF-α in LcrV-HSP70 vaccinated mice in comparison to groups vaccinated with LcrV and HSP70 alone.

Yersinia species use Type III secretion system (T3SS) to translocate effector proteins into the host target cells to breach host immune barriers (44). LcrV is an essential virulent factor that makes the tip of T3SS. Before contact with the host cells, LcrV protein is expressed on the cell membrane of *Y. pestis, enterocolitica*, and *pseudotuberculosis* (45). LcrV essentially helps in the regulation and translocation of other virulence factors into the host cell (46). It also helps Yersiniae to defeat the host immune response via IL-10 mediated immunomodulatory role by suppressing the expression of proinflammatory cytokines (47, 48). As the LcrV is a common virulence factor of *Y. pestis, enterocolitica*, and *pseudotuberculosis*, we took advantage of this to test the protective efficacy of LcrV-HSP70 against these human pathogens. However, our primary focus is to develop the subunit vaccine against plague.

Clinical symptoms of yersiniosis by *Y. enterocolitica* first appear after an incubation period of about 5 days (range 1–11 days) and include diarrhea, fever, vomiting, tenesmus, and abdominal pain. The incubation period of *Y. pseudotuberculosis* is 5–10 days; however, durations of 2–20 days have been reported in occasional outbreaks, with the average time being 4 days after exposure to the bacterium when symptoms are present (https://www.cdc.gov/yersinia/healthcare.html). Therefore, in our studies, we determined the presence of both of the pathogens in the spleen and in the blood to know whether our vaccine formulation is effective or not. We challenged all the vaccinated and control mice on day 60, and we determined the CFUs in the blood and spleen on day 61–65. On day 2 post-challenge, we observed a significant reduction in the number of CFUs in the spleen and blood of *Y. enterocolitica-* and *Y. pseudotuberculosis*-challenged mice that were immunized with LcrV-HSP70 in comparison to LcrV and HSP70 alone. Since the oral route is the natural route of infection for *Y. enterocolitica* or *Y. pseudotuberculosis*, it would be relevant in the future to determine the protective efficacy of the above vaccine candidate by a mucosal route. In the case of plague, the bivalent fusion protein LcrV-HSP70 imparts 100% protection against plague, whereas LcrV alone provided 70% protection only. There was no protection in control and HSP70-vaccinated mice. Taken together, the recombinant bivalent fusion protein LcrV-HSP70 has the scope for further evaluation of its mucosal immune response in animal models, thus increasing its potential to become a vaccine against plague and Yersiniosis.

## Data Availability Statement

All datasets generated for this study are included in the article/Supplementary Material.

## Ethics Statement

The animal study was reviewed and approved by Institutional Animal Ethics Committee (IAEC) of Defence Research and Development Establishment.

## Author Contributions

SV initiated this project and was responsible for the overall design of the study, conducting challenge experiments, interpretation of data and writing of this manuscript. AG and BN conducted the laboratory experiments. SK contributed in the interpretation of results and reviewing of this manuscript before submission.

## Conflict of Interest

The authors declare that the research was conducted in the absence of any commercial or financial relationships that could be construed as a potential conflict of interest.
