# Supplementary material for: Vaccine Potential of a Recombinant Bivalent Fusion Protein LcrV-HSP70 Against Plague and Yersiniosis
Source: Front Immunol. 2020 Jun 12;11:988. doi: 10.3389/fimmu.2020.00988 (PMC7303293; doi:10.3389/fimmu.2020.00988)
Supplement: Supplementary file 1 [file Data_Sheet_1.docx]

**Supplementary Figures**

**
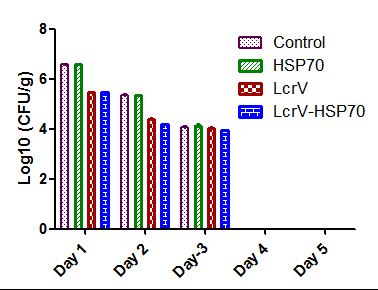
Fig. S1**

**[A]**


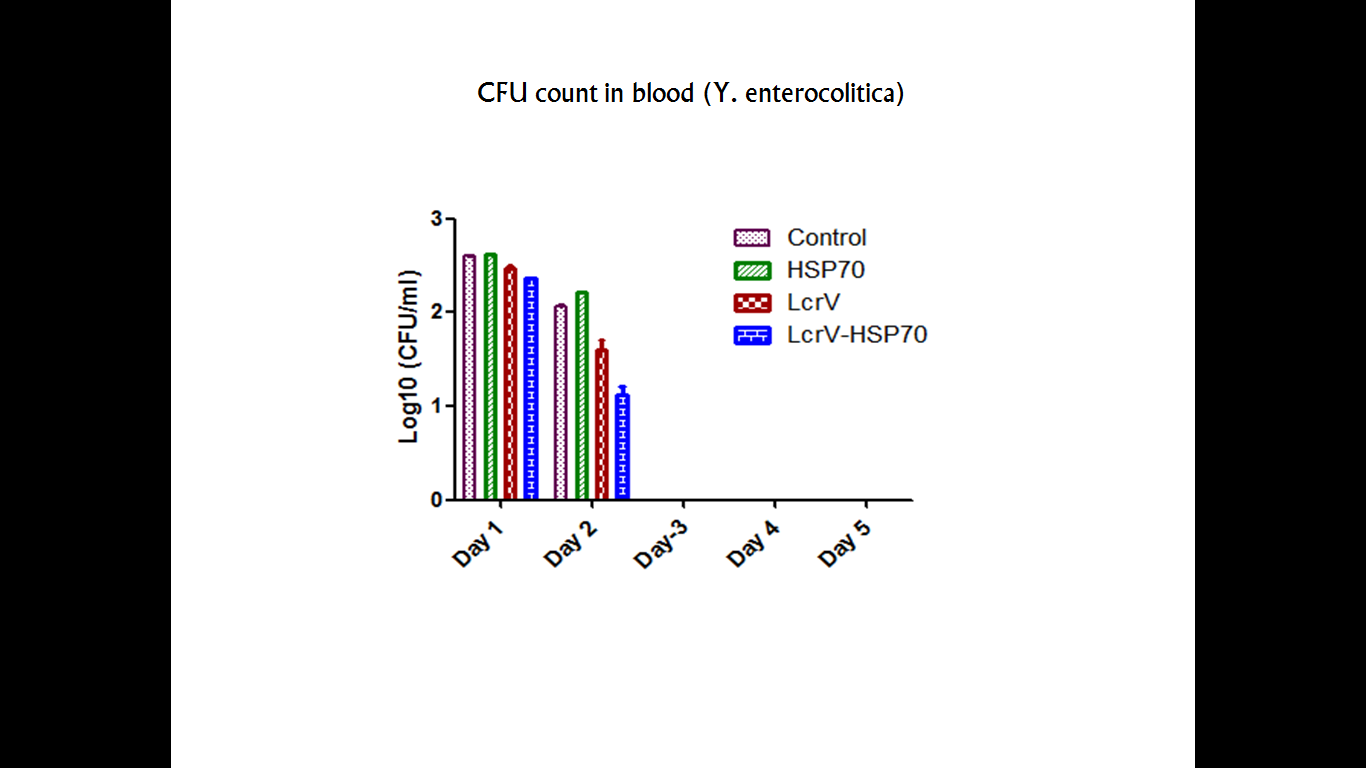


**[B]**

**
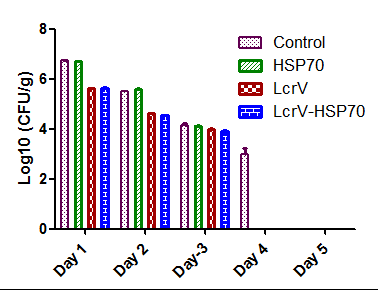
**

**[C]**

**
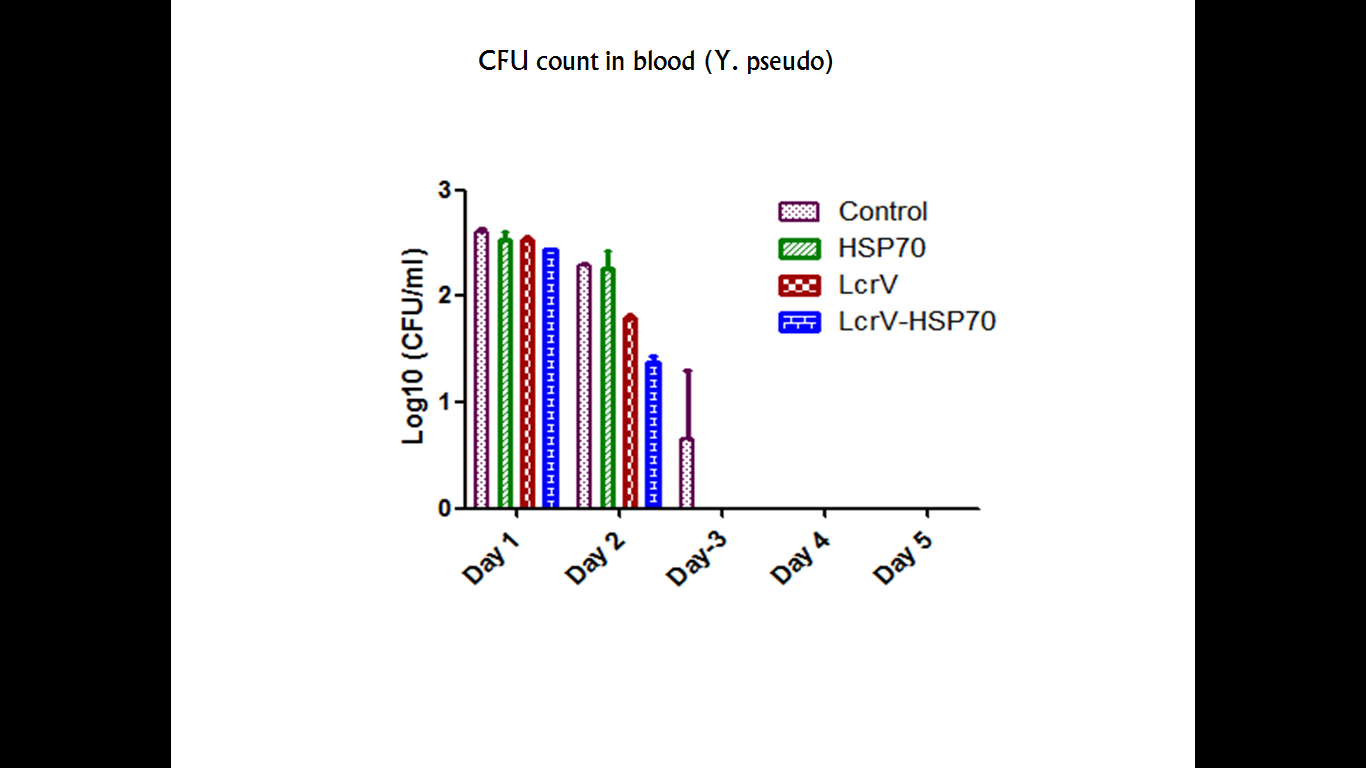
**

**[D]**

**Legend to supplementary figures**

**Figure S1.** CFU count on day 1-5 post-challenge in the spleen and blood of *Y. enterocolitica* and *Y*. *pseudotuberculosis* challenged mice: CFU count in the spleen **[A]** and blood **[B]** in *Y. enterocolitica* challenged mice. CFU count in the spleen **[C]** and blood **[D]** in *Y. pseudotuberculosis* challenged mice.
